# Therapeutic effects of mesenchymal stem cells and their derivatives in common skin inflammatory diseases: Atopic dermatitis and psoriasis

**DOI:** 10.3389/fimmu.2023.1092668

**Published:** 2023-02-20

**Authors:** Jie Yang, Minglu Xiao, Kui Ma, Hongyu Li, Mingzi Ran, Shuxu Yang, Yuguang Yang, Xiaobing Fu, Siming Yang

**Affiliations:** ^1^ Department of Dermatology, 4th Medical Centre, PLA General Hospital, Beijing, China; ^2^ Research Centre for Tissue Repair and Regeneration Affiliated to the Medical Innovation Research Department, PLA General Hospital and PLA Medical College, Beijing, China; ^3^ Tianjin Medical University, Tianjin, China

**Keywords:** MSCs (mesenchymal stem cells), MSCs derivatives, skin inflammatory diseases, atopic dermatitis, psoriasis, extracellular vesicles

## Abstract

Chronic skin inflammatory diseases including atopic dermatitis (AD) and psoriasis have been considered uncontrolled inflammatory responses, which have usually troubled patients around the world. Moreover, the recent method to treat AD and psoriasis has been based on the inhibition, not regulation, of the abnormal inflammatory response, which can induce a number of side effects and drug resistance in long-term treatment. Mesenchymal stem/stromal cells (MSCs) and their derivatives have been widely used in immune diseases based on their regeneration, differentiation, and immunomodulation with few adverse effects, which makes MSCs a promising treatment for chronic skin inflammatory diseases. As a result, in this review, we aim to systematically discuss the therapeutic effects of various resources of MSCs, the application of preconditioning MSCs and engineering extracellular vesicles (EVs) in AD and psoriasis, and the clinical evaluation of the administration of MSCs and their derivatives, which can provide a comprehensive vision for the application of MSCs and their derivatives in future research and clinical treatment.

## 1 Introduction

Skin inflammatory diseases, mainly including atopic dermatitis (AD) and psoriasis, are considered an uncontrolled response to systemic inflammation, the main symptoms and pathological features of which are manifested in the skin (1, 2). The problems caused by inflammatory skin diseases plague people all over the world and bring a huge economic burden. The incidence of AD accounts for higher than 20% of children and approximately 10% of adults in some countries including both developing and developed counties and continues to increase (3, 4). Unlike AD, psoriasis accounts for approximately 1% of children and 11% of adults in an epidemiological study of 20 countries (5). Moreover, the age distribution of psoriasis has a bimodal onset including before the age of 40 years accounting for 75% of cases and after the age of 40 years, according to two different subtypes of its pathological features (6). The reasons we choose AD and psoriasis to represent the skin inflammatory diseases are that a) AD and psoriasis are kinds of skin inflammatory diseases that affect the largest number of patients and the quality of patients’ life around the world, b) AD and psoriasis are difficult to cure and the treatments for them have a series of side effects, and c) among skin inflammatory diseases, the treatment and pathogenesis of AD and psoriasis are the hottest research topic nowadays.

As mentioned before, skin inflammatory diseases are mainly caused by the imbalance between pro- and anti-inflammatory factors. The pathogenesis of AD is known as the abnormal activation of T helper 2 (Th2) lymphocyte, which can subsequently secrete a series of pro-inflammatory cytokines including immunoglobulin E (IgE), interleukin-4 (IL-4), IL-5, IL-13, IL-17, IL-22, IL-31, and thymic stromal lymphopoietin (TSLP), leading to epidermal barrier defect and increased skin inflammation (7–9), whereas psoriasis is mainly considered the abnormal activation of Th1 and Th17 lymphocytes which secrete pro-inflammatory cytokines such as tumor necrosis factor (TNF)-α, interferon-γ (IFN-γ), IL-17, and IL-23 (10). However, although the current therapeutic methods vary from phototherapy to immunosuppressant drugs and biological agents, the curing mechanism is based on the inhibition, not regulation, of the abnormal inflammatory response to suppress the symptoms. The traditional administration of drugs, such as corticosteroids and calcineurin inhibitors, can induce a series of side effects, including absorption and hypothalamic–pituitary–adrenal axis suppression, growth suppression, atrophy, cataracts, and drug resistance in long-term treatment (11, 12). Although recent research indicates that biological agents can effectively improve the symptoms of skin inflammatory diseases, they can also induce serious side effects. For example, the JAK inhibitors including abrocitinib and dupilumab can induce a series of adverse events such as upper respiratory tract infection, conjunctivitis, asthma, and nasopharyngitis (13). The TNFα inhibitor such as adalimumab can induce adverse events such as serious infection, tuberculosis, and tumor (14).

Mesenchymal stem/stromal cell (MSC) derivatives have been widely used in clinical treatments in virtue of their abilities of regeneration, differentiation, and immunoregulation (15, 16). The resources of MSCs can be harvested from various tissues including umbilical cord (UC-MSCs) (17) or its blood (UCB-MSCs) (18), bone marrow (BM-MSCs) (19) or adipose tissue (AD-MSCs) (20), and gingiva (GMSCs) (21), which may have different therapeutic effects on skin inflammatory diseases. In addition to those resources, there are also some important issues to be considered in stem cell-based therapy, such as the number of cells transplanted, preconditioning of the cell preparation, relevant targets of the therapy, and route and frequency of administration (22–24). In addition, the MSCs of patients with skin inflammatory diseases show abnormal biological abilities to regulate inflammation, differentiation, and regeneration compared to the healthy population. The target to improving those MSCs from the patients of skin inflammatory diseases may become another cured target for clinical treatments (25–27). However, there are still mild adverse effects such as headache, fever, and the risk of embolism, but there have been no documented cases of embolism during treatment of AD and psoriasis patients (28, 29). It is worth noting that various administrations of MSCs and their derivatives may have obviously different effects on treating common chronic skin inflammatory diseases, AD, and psoriasis. As a result, in this review, we provide an overview of current strategies regarding the use of MSC derivatives including the therapeutic effects of different resources, preconditioning of the cell preparation, extracellular vesicles (EVs), and the improvements of MSCs in the lesion skin; a clinical evaluation of patients treated who have MSCs in inflammatory skin diseases; and future directions needed to develop this field.

## 2 The therapeutic target aiming at lesional MSCs in AD and psoriasis

**Figure 1:**
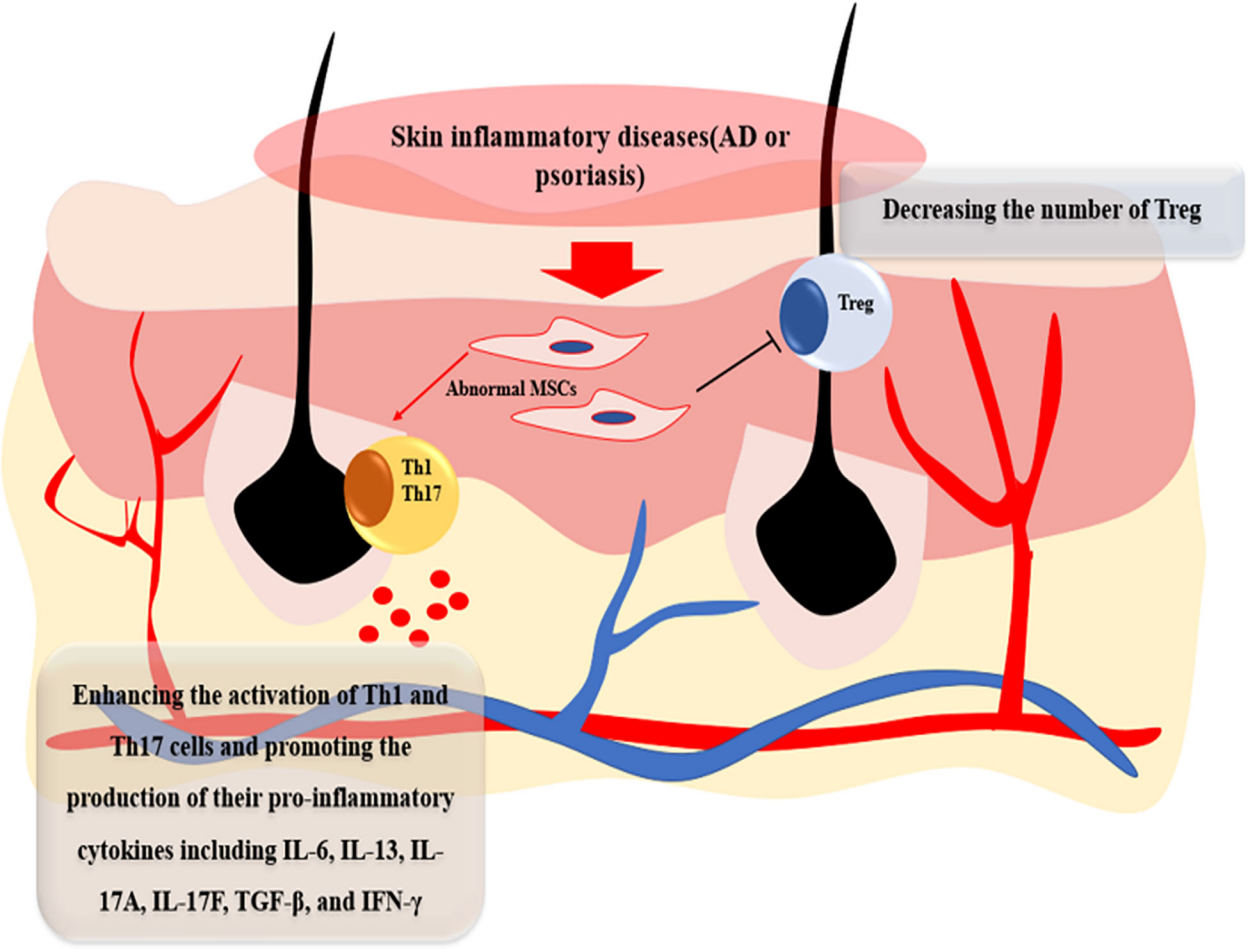
The effects of lesional MSCs in AD and psoriasis patients. Compared with MSCs from healthy donor, MSCs in skin lesion of AD and psoriasis patients can enhance the activation of Th1 and Th17 cells and promote the production of their pro-inflammatory cytokines including IL-4, IL-6, IL-13, IL-17A, IL-17F, IL-17C, CCL, TNF-α, and IFN-γ and suppress the activation of Treg.

AD and psoriasis are systemic and immune-allergic inflammatory skin diseases, the mechanism of which is the dysregulation of immunology (30–32), whereas MSCs play a role on regeneration and more importantly on immunomodulation (33–35). As a result, it could be the new clinical target whether the biological function of MSCs in skin lesion of AD and psoriasis changed and evolved in the pathogenesis of skin inflammatory diseases. Recent studies reveal that skin-derived MSCs in patients show an obviously differential function compared with common MSCs. Orciani et al. found that MSCs isolated from the skin lesion of patients in AD can enhance the activation of Th1 and Th17 cells and promote the production of their pro-inflammatory cytokines including IL-6, IL-13, IL-17A, IL-17F, transforming growth factor-beta (TGF-β), and IFN-γ, whereas they decrease the number of Th2 cells and their production including IL-2, IL-4, IL-5, and IL-23A. Interestingly, some proinflammatory factors are not changed including chemokine (C–C motif) ligand 1 (CCL1), IL-17C, and TNF-α (36). Campanati et al. also found that MSCs derived from patients of AD can overexpress the levels of IL-6 and IL-13 whereas there is no significance with the level of IL-4 compared with healthy MSCs (27). In psoriasis, recent research found that MSCs from skin lesion performed an abnormal role in two ways compared with normal MSCs. Firstly, compared with healthy donors, MSCs from skin lesion of psoriasis patients decreased the level of TGF-β and its receptor and thus increased the ratio of Th17/Treg and their inflammatory cytokines including IFN-γ and TNF-α (37–40). Secondly, pathological MSCs from psoriasis patients expressed high levels of vascular endothelial growth factor (VEGF) and inducible nitric oxide synthase (iNOS), which was different from the MSCs of the normal population and AD patients. The increasing levels of VEGF and iNOS would be more vulnerable to recruit a number of immune cells, proinflammatory cytokines, and chemokines into skin lesion (41–43). Moreover, as AD and psoriasis are a kind of immune diseases, the hematopoietic microenvironment is altered in those chronic inflammatory diseases. Compared with BM-MSCs from healthy people, BM-MSCs show an abnormal secretion of inflammatory cytokines and chemokines in patients with psoriasis, which showed a different hematopoietic microenvironment (26). In addition, Zhang et al. found that bone marrow hematopoietic stem cells (BMHSCs) from psoriasis patients have a different cell phenotype and an increased expression of CD45, which may account for the activation of T cells and be closely associated with disease severity (44) (**Figure 1**). Considering that the inflammatory cascade in AD and psoriasis begins at the mesenchymal level, an upstream therapeutic intervention to treat the abnormal MSCs can potentially improve the pathogenesis of those inflammatory diseases. However, the therapeutic methods to treat lesional MSCs have been still unrevealed and how to use the improved MSCs to treat the skin inflammatory diseases needs to be further studied.

## 3 The effects of MSCs from different resources on AD and psoriasis

With advancing technology for administering MSCs, the application of MSCs has emerged as a promising strategy for the treatment of skin inflammatory disease due to their capability of regeneration, immunomodulation, and differentiation (45). Recent research found that MSCs have efficacy in the reduction of disease severity and epidermal thickness, arranging layers of epidermal layers, and keeping an intact basement membrane through its powerful capability of immunoregulation (46, 47). MSCs can be harvested from different tissues as mentioned before, whereas from different resources MSCs may have various therapeutic effects on skin inflammatory diseases.

### 3.1 AD-MSCs

Among various resources of MSCs, AD-MSCs have become one of the most attractive therapies because of their easy way to harvest, few ethical concerns, and most importantly their secreting capacity of numerous growth factors and adipokines to assist tissue survival (48). In AD, intravenous administration of human AD-MSC in mice (2 × 10^5^ or 2 × 10^6^ cells/200 μL normal saline) can alleviate allergic inflammation which includes decreasing the number of degranulated mast cells (MCs), IgE level, amount of histamine released, and prostaglandin E2 level; inhibiting the secretion of pro-inflammatory cytokines and chemokines; increasing the expression of Th1 and Th2 cells; and promoting the expression of regulatory T (Treg) cells (49). Kim et al. found that intravenous administration of AD-MSCs in mice [1 × 10^6^ cells in 100 µl phosphate-buffered saline (PBS)] can decrease the macrophage inflammatory protein-2 (MIP-2) level to overexpress the miR-122a-5p level, regulating the level of cytokine signaling 1 (SOCS1), to decrease the internal inflammation and clinical symptoms (20). Except for the beneficial effects mentioned previously, Guan et al. also found that subcutaneous injection of mouse AD-MSCs in mice (1 × 10^6^ cells in 1ml PBS) can especially inhibit the expression of Th17 and its relative pro-inflammatory products including IL-17A, CCL20, and matrix metalloproteinase 12 (MMP12) in AD (50) (**Table 1**).

**Table 1:** The therapeutic effects of MSCs and their derivatives from different sources in AD.

	Sources of MSCs	Animal model	Route of administration	Dose	Main outcome
Shin et al., 2017 (49)	Human AD-MSCs	Mouse model induced by *Dermatophagoides farinae*	Intravenous	2 × 10^5^/2 × 10^6^ cells	hAD-MSCs reduced epidermal thickness, lymphocyte infiltration, and MC degranulation
Kim et al., 2018 (22)	Human AD-MSCs	Mouse model induced by using DNCB	Intravenous	1 × 10^6^ cells on 12 and 23 days	Decreasing MIP-2 to overexpress the level of miR-122a-5p, regulating the level of cytokine signaling 1 (SOCS1), to decrease the internal inflammation and clinical symptoms
Guan et al., 2022 (50)	Mouse AD-MSCs	Mouse model induced by ovalbumin	Subcutaneous	1 × 10^6^ cells	Inhibiting the expression of Th17 and its relative pro-inflammatory products
Park et al., 2020 (18)	Human UCB-MSCs	Mouse model induced by *Dermatophagoides farinae*	Subcutaneous	2 × 10^6^ cells	Decreasing the level of TNFα to inhibit the infiltration of MC and decrease the level of IgE into skin lesions by secreting transforming growth factor-beta (TGF-β)
Shin et al., 2021 (28)	Human UCB-MSCs	Mouse model induced by *Dermatophagoides farinae*	Subcutaneous	2 × 10^6^ cells	Reducing allergic inflammatory symptoms by inhibiting Th2 cell differentiation and MC activation through the COX2–PGE2 pathway
Jung et al., 2022 (51)	Human UCB-MSCs	Mouse model induced by *Dermatophagoides farinae*	Subcutaneous	2 × 10^6^ cells	Decreasing IL-4, TNF-α, TARC, and IL-22 through EGF in skin lesion
Jung et al., 2021 (52)	Human TMSCs	Mouse model induced by using DNCB	Subcutaneous	2 × 10^4^ cells	Decreasing IL-6, IL-1β, TNF-α, and IL-4 secreted by Th1 and Th2 cells, respectively, and IgE secreted by B cells and MC
Na et al., 2014 (19)	Mouse BM-MSCs	Mouse model induced by ovalbumin	Intravenous	2 × 10^5^ cells	Suppressing T cells and its inflammatory products by NO-dependent pathways. Suppressing B cells and IgE by the downregulation of AID and BLIMP-1.
Xiong et al., 2022 (53)	Human sheds	Mouse model induced by using DNCB	Intravenous/subcutaneous	2 × 10^7^ cells/mL, and 2 × 10^6^ cells on days 17, 24, and 31	Improving the disruption of skin barrier function and enlarged spleens. Decreasing IgE and TLSP Inhibiting the activation of Th1, Th2, and Th17 cells in skin lesion
Sah et al., 2018 (54)	Human UCB-MSCs (SOD3-tranduced)	Mouse model induced by ovalbumin	Subcutaneous	2 × 10^6^ cells on days 20, 28, and 42	Alleviating the allergic inflammation in keratinocytes through competitively interacting with H4R and IL-4Rα. Reducing the inflammation in the skin through the JAK-STAT pathway
Park et al., 2019 (55)	Human WJ-MSCs (preconditioned with the TLR3 agonist poly I:C or IFN-γ)	Mouse model induced by Af extract	Subcutaneous	\	Decreasing proinflammatory cytokines. Ameliorating epidermal thickness and inflammatory cell infiltration in skin lesions.
Cho et al., 2018 (56)	Human AD-MSCs (EVs)	Mouse model induced by *Dermatophagoides farinae*	Intravenous/subcutaneous	0.14, 1.4, and 10 μg/head	Reducing pathological symptoms, serum IgE, the number of eosinophils in blood, and the infiltration of MC, CD86+, and CD206+ cells in skin lesions. Decreasing IL-4, IL-23, IL-31, and TNF-α in AD skin lesions
Shin et al., 2020 (57)	Human AD-MSCs (EVs)	Mouse model induced by oxazolone (Ox)	Subcutaneous/topical	1, 3, and 10 μg/head	Restoring epidermal barrier functions in AD by facilitating the *de novo* synthesis of ceramides
Kim et al., 2022 (58)	Canine AD-MSCs (EVs)	Mouse model induced by using DNCB	Subcutaneous	2 × 10^10^ particles/head	Decreasing serum IgE, epidermal inflammatory cytokines, such as IL-4, IL-13, IL-31, RANTES, and TARC. Repairing skin barrier by restoring transepidermal water loss, enhancing stratum corneum hydration, and upregulating the expression levels of epidermal differentiation proteins. Reducing IL-31/TRPA1-mediated pruritus and activation of JAK/STAT signaling pathway

### 3.2 UCB-MSCs

Another type of MSCs only found in treating AD is from umbilical cord blood. UCB-MSCs have the advantage of having an easy way to harvest and a low probability of pathophoresis (59). In AD, three ways are found in immunomodulatory effects functioned by UCB-MSCs in recent research. The first one is that subcutaneous administration of UCB-MSCs with 2 × 10^6^ cells in mice can decrease the level of TNFα to inhibit the infiltration of mast cells and decrease the level of IgE into skin lesions by secreting TGF-β (18). Second is that subcutaneous administration of UCB-MSCs in mice (2 × 10^6^ cells) can reduce allergic inflammatory symptoms by inhibiting Th2 cell differentiation and mast cell activation through the cyclooxygenase-2 (COX2)–prostaglandin E2 (PGE2) pathway (60). The last is that subcutaneous administration of UCB-MSCs in mice (2 × 10^6^ cells) can decrease the level of pro-inflammatory cytokines including IL-4, TNF-α, thymus, activation-regulated chemokine (TARC), and IL-22 through secreting the epidermal growth factor (EGF) in skin lesion (61) (**Table 1**).

### 3.3 UC-MSCs

On the contrary, research on the effects of UC-MSCs in psoriasis has attracted extensive attention, whereas little attention is given to those in AD. In psoriasis, subcutaneous or intravenous administration of UC-MSCs (2 × 10^6^ cells) can effectively reduce the severity of psoriasis-like dermatitis, delay the appearance of skin lesions, and accelerate the recovery of skin lesions by reducing the number of Th1 and Th17 cells and their secreted pro-inflammatory products and increasing the number of Treg cells (17). Other research found that intravenous administration of UC-MSCs in mice (1 × 10^6^ cells) can inhibit the infiltration of immune cells into the dermal layer and suppress the secretion of IFN-γ from plasmacytoid dendritic cells (pDCs) (52).

### 3.4 MSCs from other resources

Despite fewer applications of other resources of MSCs in recent research, they indeed play an important role in treating skin inflammatory diseases. Unlike AD-MSCs or BM-MSCs, which include invasive procedures to harvest the cells, inadequate numbers for production, and the most worrying problem, that is, the sources from the mesoderm may have barriers to differentiation into ectodermal and endodermal tissues (55, 62), tonsil-derived mesenchymal stem cells (TMSCs) can be easily isolated from surgically removed tonsil and expanded in cultures, which have been proposed as an alternative source of adult stem cells (63). In AD, subcutaneous administration of TMSCs in mice (2 × 10^4^ cells) can decrease the levels of pro-inflammatory cytokines including IL-6, IL-1β, TNF-α, and IL-4 secreted by Th1 and Th2 cells and the level of IgE secreted by B cells and mast cells (56). Bone marrow is the classical resource to harvest MSCs, but it is limited in production—BM-MSCs have been to some extent in AD. Na et al. found that intravenous administration of BM-MSCs in mice (2 × 10^5^ cells) can suppress the activation of T cells and B cells. The T cell and its inflammatory products including IFN-γ and IL-4 have been suppressed by nitric oxide (NO)-dependent pathways to increase the level of transcription factors including T-bet, GATA-3, and c-Maf. B cells and IgE have been suppressed by the downregulation of AID and BLIMP-1 (19). Interestingly, some sources from the oral cavity to harvest MSCs have been found in treating AD and psoriasis. Xiong et al. found that subcutaneous or intravenous administration of MSCs isolated from human exfoliated deciduous teeth (SHEDs), a special type of MSCs with superior capability of immunoregulation, in mice (2 × 10^6^ cells) can effectively improve the disruption of skin barrier function and enlarged spleens, decrease the levels of IgE and TLSP, and inhibit the activation of Th1, Th2, and Th17 cells in skin lesion (57). Moreover, Ye et al. found that intravenous administration of human gingiva-derived MSCs in mice (2 × 10^6^ cells) can reduce the levels of pro-inflammatory cytokines including IFN-γ, TNF-α, IL-6, IL-17A, and IL-21 secreted by Th1 and Th17 cells and promote the increasing number of Treg cells in mouse psoriasis-like models (21). However, studies on the application and comparison of different types of MSCs in skin inflammatory diseases are still lacking. As a result, different types of MSCs in therapeutic effects of MSCs from the various resources in AD and psoriasis need to be further elucidated (**Tables 1**, **2**).

**Table 2:** The therapeutic effects of MSCs and their derivatives from different sources in psoriasis.

	Sources of MSCs	Animal model	Route of administration	Dose	Main outcome
Chen et al., 2022 (17)	Human UC-MSCs	Mouse model induced by IMQ cream	Intravenous/subcutaneous	2 × 10^6^ cells	Reducing the severity of psoriasis-like dermatitis. Delaying the appearance of skin lesions. Accelerating the recovery of skin lesions by reducing the number of Th1 and Th17 cells and their secreted pro-inflammatory products. Increasing the number of Treg cells.
Chen et al., 2019 (64)	Human UC-MSCs	Mouse model induced by IMQ cream	Intravenous	1 × 10^6^ cells	Inhibiting the infiltration of immune cells into the dermal layer Suppressing the secretion of IFN-γ from pDCs
Ye et al., 2022 (21)	Human gingiva-derived MSCs	Mouse model induced by IMQ cream	Intravenous	2 × 10^6^ cells on 1 and 4 days	Reducing pro-inflammatory cytokines including IFN-γ, TNF-α, IL-6, IL-17A, IL-21 secreted by Th1 and Th17 cells Promoting the number of Treg cells
Sah et al., 2016 (65)	Human UC-MSCs (SOD3-tranduced)	Mouse model induced by IMQ cream	Subcutaneous	2 × 10^6^ cells	Ameliorating the symptoms of skin lesion by regulating the inflammatory pathways including TLR-7, NFκB, MAPK, and JAK-STAT pathways
Meng et al., 2021 (66)	Human DPSCs (HGF-transduced)	Mouse model induced by IMQ cream	Intravenous	2 × 10^6^ cells	Ameliorating the psoriasis-like erythema, scaling, and thickening and the expression of CK6 and CK17. Decreasing inflammatory cytokines such as IFN-γ, IL-6, and TNF-α. Reducing the number of Th1. Increasing the number of Th2.i90op.
Zhang et al., 2022 (67)	Human UC-MSCs (IFNγ-sEVs)	Mouse model induced by IMQ cream	Intradermal	150 µg	Reducing the symptom of psoriasis through decreasing the levels of pro-inflammatory cytokines including IL-17A, IFN-γ, IL-6, and TNF-α and Th17 cells. Increasing the population of Th2 cells in both spleen and skin.
Zhang et al., 2022 (68)	Human UC-MSCs (EVs)	Mouse model induced by IMQ cream	Subcutaneous	50 µg	Reducing proinflammatory cytokines and chemokines including IL-17, IL-23, TNFα, and CCL20 suppressing the activation of DCs through inhibiting the JAK-STAT pathway

## 4 The therapeutic effects of preconditioning MSCs on AD and psoriasis

**Figure 2:**
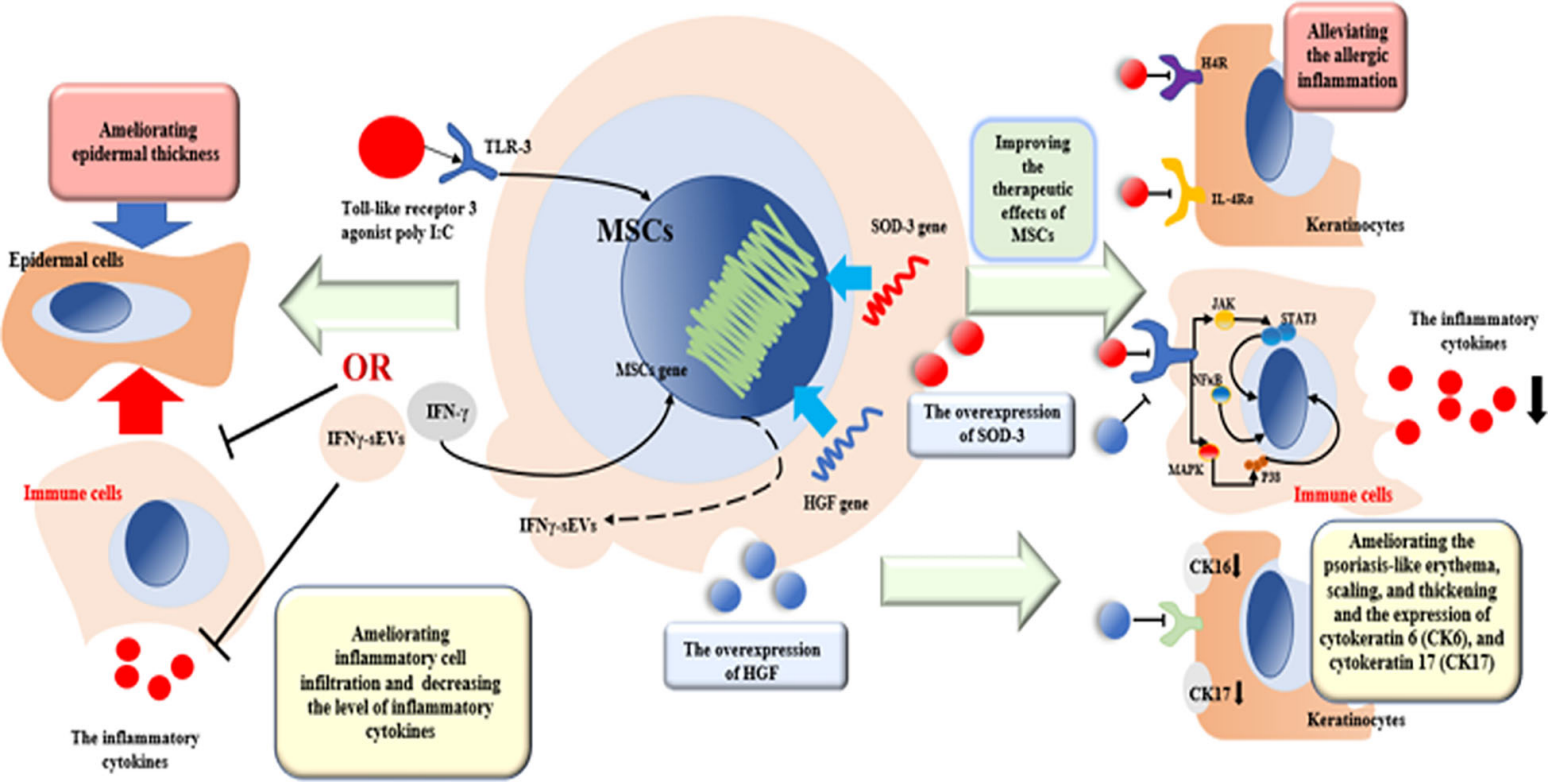
The potential mechanism of preconditioned MSCs and EVs in treating AD and psoriasis. SOD3-transduced MSCs can alleviate the allergic inflammation in keratinocytes through competitively interacting with H4R and IL-4Rα. SOD-MSCs can reduce the inflammation in the skin through the NFκB, MAPK, and JAK-STAT pathways. HGF-transduced MSCs can ameliorate psoriasis-like erythema, scaling, and thickening and the expression of CK6 and CK17 and decrease pro-inflammatory cytokines such as IFN-γ, IL-6, and TNF-α. Preconditioned with the TLR3 agonist poly I:C or IFN-γ can ameliorate more epidermal thickness and inflammatory cell infiltration in skin lesions. IFNγ-sEVs can decrease pro-inflammatory cytokines including IL-17A, IFN-γ, IL-6, and TNF-α and Th17 cells and increase the population of Th2.

With the technology advancing, recent research generally indicates that preconditioning MSCs can effectively improve the immunoregulation capability in treating diverse immune diseases (51, 64, 69). Superoxide dismutase (SOD), an antioxidant enzyme, plays an essential role in inflammatory diseases, which can convert the superoxide to hydrogen peroxide and oxygen and exert an anti-inflammatory role (70). SOD3, an extracellular isoform of SOD, can be transduced into MSCs, which can increase the therapeutic potency of MSCs in antioxidant response and immunomodulation (53). In AD, Sah et al. found that subcutaneous administration of SOD3-transduced UCB-MSCs (SOD-MSCs) in mice (2 × 10^6^ cells) can improve the therapeutic effects of MSCs in two pathways. Firstly, SOD-MSCs can alleviate the allergic inflammation in keratinocytes through competitively interacting with the histamine H4 receptor (H4R) and IL-4Rα. Secondly, SOD-MSCs can reduce the inflammation in the skin through the JAK-STAT pathway (54). In psoriasis, another research found that subcutaneous administration of SOD-MSCs (UC-MSCs) in mice (2 × 10^6^ cells) can ameliorate the symptoms of skin lesion by regulating the inflammatory pathways including toll-like receptor-7, nuclear factor-kappa B(NFκB), p38 mitogen-activated kinase (MAPK), and JAK-STAT pathways (71) (**Figure 2**). Other studies indicate that hepatocyte growth factor-transduced MSCs (HGF-MSCs) also exert a better capability of antioxidant response in various acute/chronic disease (58). Meng et al. found that in psoriatic skin lesions, intravenous administration of HGF-transduced dental pulp stem cells (HGF-DPSCs) in mice (2 × 10^6^ cells) can ameliorate psoriasis-like erythema, scaling, and thickening and the expression of cytokeratin 6 (CK6) and cytokeratin 17 (CK17). In addition, in inflammatory respect, HGF-DPSCs can decrease the levels of inflammatory cytokines such as IFN-γ, IL-6, and TNF-α; reduce the number of Th1; and increase the number of Th2. Moreover, HGF-DPSCs exerts more efficacy compared with pure DPSCs (72) (**Figure 2**). Finally, preconditioning MSCs with inflammatory factors can more effectively treat skin inflammatory diseases. Park et al. revealed that subcutaneous administration of human Wharton’s jelly-derived MSCs (WJ-MSCs) preconditioned with the Toll-like receptor 3 agonist poly I:C or IFN-γ can decrease the levels of proinflammatory cytokines in a murine model of AD. Moreover, they can ameliorate more epidermal thickness and inflammatory cell infiltration in skin lesions than non-preconditioned MSCs (73). Zhang et al. found that intradermal administration of EVs derived from UC-MSCs stimulated by IFN-γ (IFNγ-sEVs) (150 µg) in mice can effectively reduce the symptom of psoriasis through decreasing the levels of pro-inflammatory cytokines including IL-17A, IFN-γ, IL-6, and TNF-α and Th17 cells and increasing the population of Th2 cells in both spleen and skin in psoriasis (74) (**Figure 2**). Up to now, there are still few research focusing on preconditioning MSCs in AD and psoriasis, which need to be further studied.

## 5 The therapeutic effects of EVs derived from MSCs on AD and psoriasis

Although MSCs have been widely used for treating skin inflammatory diseases due to their immunomodulation capability, but an increasing concern about their adverse effects such as embolism and inefficient homing to the target limits their application. Recent research revealed that EVs derived from MSCs cannot only effectively be instead of the functions of MSCs but also easy to be engineered to better exert their functions (17, 65, 74, 75). In AD, EVs are mainly from AD-MSCs and function in three ways. Firstly, in the AD mouse model, subcutaneous administration of EVs derived from AD-MSCs in mice can reduce the levels of inflammatory cytokines and IgE including IL-4, IL-5, IL-13, TNF-α, IFN-γ, IL-17, and TSLP in skin lesion. Secondly, they can reduce trans-epidermal water loss and enhance stratum corneum (SC) hydration. At last, EVs can restore the skin barrier and lipid metabolism in skin lesion (66, 67, 76). Moreover, in psoriasis, Zhang et al. found that subcutaneous administration of EVs derived from UC-MSCs in mouse psoriasis-like models (50 µg) can effectively reduce the level of proinflammatory cytokines and chemokines including IL-17, IL-23, TNFα, and CCL20 and suppress the activation of DCs through inhibiting the JAK-STAT pathway (68). Another research found that intradermal administration of IFNγ-preconditioned EVs from UC-MSCs in mice (150 µg) can also reduce the symptoms of psoriasis described previously (74) (**Figure 2**). However, the different resources and mechanism of EVs are still under revealed and the engineered EVs are little applied in treating skin inflammatory diseases, which needs to be further studied in future.

## 6 The clinical efficacy of MSCs and their derivatives applied in AD and psoriasis

The administration of MSCs in AD and psoriasis has been tested in clinical treatment; the efficacy and side effects are detailed in the following part. Shin et al. intravenously administered BM-MSCs to five patients with AD (1.0 × 10^6^ cells/kg three times every 2 weeks) and observed for 16 weeks (treatment for 4 weeks and follow-up for 12 weeks). After 16 weeks, the follow-up was to identify the period during which the patient’s improved symptoms are maintained by using medications without additional use of systemic steroids and immunomodulators. They found that the Eczema Area and Severity Index (EASI) improved significantly at 16 weeks, which had a long-term efficacy for an average of 38 weeks (range, 16–86), whereas it showed no serious side effects in the patients. Moreover, the pro-inflammatory cytokines in their blood significantly decreased at the end point (28). Similarly, Kim et al. recruited 34 patients with moderate-to-severe AD with a follow-up for 1 and 3 months revealed that subcutaneously administering a high dose of UCB-MSCs (5.0 × 10^7^ cells) could effectively reduce the symptom of AD and with little adverse effects and no relapse (29). In psoriasis, Ahn et al. found a clinical case that a 47-year-old man, diagnosed with psoriasis in 1995, has received various treatments for 25 years but with no improved psoriatic condition. After both intravenous and local administration of UC-MSCs, his erythema gradually disappeared (10 ml for intravenous administration with 3 × 10^6^ cells/ml in 0.9% physiological saline and 2–4 ml for local administration with 1 × 10^6^ cells/ml in 0.9% physiological saline). The follow-up was 122 days, and the symptom was gradually becoming better without any side effects (77). In addition, in a 17-patient clinical trial, Cheng et al. also found that intravenous administration of UC-MSCs (1.5 × 10^6^ cells/kg once time every 2 weeks, four times as a course of treatment) can effectively reduce the symptoms of psoriasis with no severe adverse effects. The follow-up was at 15 days, 30 days, 45 days, 2 months, 3 months, and 6 months after treatment and there are no relapse and severe adverse effects observed (78). However, the number of clinical studies of using MSCs in AD and psoriasis is still inadequate. Moreover, more detailed information such as the choosing of safe dose, resources, and delivery way of MSCs should be cleared, which needs to be further studied.

## 7 Comparing alternatives of MSCs and their derivatives applied in AD and psoriasis

There are some limitations in the application of MSCs and their derivatives: a) there is a need for the conduction of double-blinded, placebo-controlled studies to indicate the potential clinical application of MSCs in AD and psoriasis, and b) the production and cost of MSCs cannot reach the standard, which makes it difficult to translate into clinical treatment. However, the application of those treatments is still more effective in treating AD and psoriasis at the present compared with other treatments. In the application of different resources of MSCs in both AD and psoriasis, we have summarized the mechanism of different types of MSCs in the diseases. Interestingly, we found that UC-MSCs performed in the present studies were only used in psoriasis (17, 52, 78), UCB-MSCs only in AD (18, 59–61). It may be attributed to the mechanism of both kinds of MSCs to regulate inflammation, in which UC-MSCs were more likely to regulate the activation of Th1 and Th17 cells and their production, whereas UCB-MSCs were more likely to regulate the Th2 cells as present studies have mentioned. However, there is no research to compare both kinds of MSCs in the same skin inflammatory diseases. Moreover, various resources of MSCs were applied to administer into AD such as AD-MSCs, UCB-MSCs, TMSCs, BM-MSCs, and MSCs from the oral cavity, but only UC-MSCs were directly used in psoriasis in the present studies as mentioned above. Firstly, it may be attributed to the different pathogenesis of AD and psoriasis (79), whereas both the two diseases show signs of dysregulation of inflammation (80). Secondly, the chosen resources of MSCs may be primarily biased on the local storage facilities and policies of MSCs (the application of UC-MSCs in psoriasis mainly from Chinese). However, the comprehensive and systematic comparison of MSCs lines is still urgently needed in AD and psoriasis at the present.

Despite many studies associated with the application of adult mesenchymal stem cells such as AD-MSCs in treating AD and psoriasis, there are still limitations. Firstly, the adult-MSCs can just be harvested from the patients, which may limit the production of MSCs. Secondly, the therapeutic effects of MSCs can be seriously influenced by the age of the patients. Lastly, as we mentioned in the manuscript, the MSCs from patients may have lower effects compared with healthy donors, whereas compared with adult MSCs, MSCs from the fetus such as UC-MSCs have lower immunogenicity and more powerful therapeutic effects. Most importantly, they can be extracted from oneself or non-relative donators, thus enhancing the production of MCSs. As a result, we believe that MSCs from the fetus such as UC-MSCs may be the best resource to be employed to treat AD and psoriasis.

Furthermore, based on the present studies in which the accurate target is still unrevealed, the preconditioned MSCs showed more healing capability than normal MSCs as mentioned in the previous paragraphs, which makes the preconditioning technology a more promising method in dealing with MSCs *in vitro*—the next step in choosing the resource of MSCs. Above all, the two skin inflammatory diseases have also been accompanied by other immune diseases, aside from skin symptoms (81–84). Based on the condition, only subcutaneous injection of MSCs to improve the skin symptoms cannot be enough to cure the AD and psoriasis completely. Moreover, intravenous injection of MSCs increases the risk of embolism. As a result, administration of a small molecule, EVs from MSCs, through the vein may be the best method to avoid the side effects. Moreover, the present studies uncovered that EVs from MSCs can be almost a substitute of MSCs in treating diseases, but EVs alone cannot accurately home to the target of the diseases. As mentioned above, engineered EVs with the targeted ligand may be perfect to resolve the problem. However, the technology applied to engineer EVs has been little studied and there is still a need to find out the key targets of AD and psoriasis. Moreover, despite the advancing technology and that the application of MSCs has been widely used in clinical treatment, the price for the administration is still high beyond the expectation of patients, not to mention using biological programming techniques in engineering EVs from MSCs to treat skin problems. Another strategy for the application of MSCs is subcutaneous administration of MSC-CM (conditioned medium), which may dissolve the high cost of MSCs (85). However, as the content of MSC-CM may not be ensured, the effects including therapeutic efficacy and adverse effects and its mechanism need more studies to elucidate. Above all, the techniques to improve the production of MSCs and thus decrease the cost will not only stimulate more and more studies on MSCs in treating diseases but also allow more patients around the world to use MSCs and their derivatives to improve their refractory disease.

## 8 Conclusion

Chronic skin inflammatory diseases such as AD and psoriasis are mainly caused by unregulated immune response, which not only can induce the symptoms of skin lesion but also are accompanied by other immune diseases. Evidence of therapeutic effects and mechanisms found by current studies indicates that biological therapy based on MSCs and their derivatives is a promising approach for the treatment of skin inflammatory diseases. As a result, additional studies aiming at uncovering the mechanisms of the therapeutic effects of MSCs in AD and psoriasis may help define better therapeutic strategies for these diseases.

## Author contributions

SY, JY, KM, and XF conceived and designed the review. JY, MX, HL, MR, ShY and YY prepared the figures. JY, HL, SY, and XF wrote the manuscript. All authors contributed to the article and approved the submitted version.
